# Searching for serum biomarkers linking coronary heart disease and *Helicobacter pylori* infection using infrared spectroscopy and artificial neural networks

**DOI:** 10.1038/s41598-022-23191-z

**Published:** 2022-10-31

**Authors:** Weronika Gonciarz, Łukasz Lechowicz, Mariusz Urbaniak, Tomasz Rechciński, Maciej Chałubiński, Marlena Broncel, Wiesław Kaca, Magdalena Chmiela

**Affiliations:** 1 Department of Immunology and Infectious Biology, Faculty of Biology and Environmental Protection, University of Lodz, Banacha 12/16, 90-237 Lodz, Poland; 2 Departament of Microbiology, Faculty of Natural Sciences, Jan Kochanowski University, Świętokrzyska 11, 25-406 Kielce, Poland; 3 Department of Synthesis and Structural Research, Faculty of Natural Sciences, Jan Kochanowski University, Świętokrzyska 11, 25-406 Kielce, Poland; 4 Clinic and Department of Cardiology, Medical University of Lodz, 92-213 Lodz, Poland; 5 Department of Immunology and Allergy, Medical University of Lodz, Pomorska 251, 91-347 Lodz, Poland; 6 Laboratory of Tissue Immunopharmacology, Department of Internal Diseases and Clinical Pharmacology, Medical University of Lodz, Kniaziewicza 1/5, 91-347 Lodz, Poland

**Keywords:** Autoimmunity, Clinical microbiology

## Abstract

*Helicobacter pylori* (Hp) Gram-negative bacteria cause gastritis or gastric ulcers. They may be involved in the development of systemic diseases i.e. coronary heart disease (CHD). Both Hp infection and CHD are related to inflammation accompanied by C-reactive protein (CRP), tumor necrosis factor alfa (TNF-α) and homocysteine. Low density lipoprotein (LDL) and triglicerides are a classic risk factors of CHD. Infrared spectroscopy has been introduced for monitoring chronic infections or endogenous disorders using specific absorption bands for biocomponents typed as diagnostic markers. In this study we selected specific motives of infrared radiation (IR) spectra for the sera from CHD patients infected with Hp. In total 141 sera were used: 90 from patients with CHD, all Hp positive, and 51 from healthy donors, 32 Hp negative and 21 Hp positive. Hp status was evaluated by anti-Hp IgG antibodies and/or ^13^C urea breath testing. IR spectra were measured using FT-IR/FT-NIR Spectrum 400 spectrometer (PerkinElmer) chemometrically analyzed using artificial neural networks and they showed differences in absorption bands corresponding to triglicerides, CRP, homocysteine, LDL and TNF-α, and selected component groups between CHD patients infected with Hp vs healthy uninfected donors (96.15% accuracy). Triglicerides and CRP were the best biomarkers linking Hp infection with CHD.

## Introduction

Coronary heart disease (CHD) is a chronic disease of the coronary vessels, which is classified as inflammatory disease^1–3^. This is because of an increased level of inflammatory cytokines such as tumor necrosis factor (TNF)-α, interleukin (IL)-6, upregulation of adhesion molecules: intracellular adhesion molecules (ICAM)-1 and -2, vascular cell adhesion molecule 1 (VCAM-1), platelet endothelial cell adhesion molecule 1, macrophage chemotactic protein-1 (MCP-1), P-,L-,E- selectins, and acute phase proteins including C-reactive protein (CRP), amyloid A, fibrinogen and homocysteine^4–9^. Classic risk factors of CHD include, hypertension, elevated levels of total cholesterol, triglycerides and low density lipoprotein (LDL) vs decreased level of high density lipoprotein (HDL), diabetes mellitus, raised homocysteine and coagulation factors as well as nicotinizm^10–12^.

In numerous studies the relationship between CHD and infectious agents, including bacterial pathogens such as *Chlamydophila pneumoniae, Mycoplasma pneumoniae, Helicobacter pylori*, periodontal pathogens and among viruses, *Cytomegalovirus* and *Herpes simplex* virus has been suggested. These microorganisms may occur in atherosclerotic plaques or may stimulate local and systemic inflammation and thus influence atherogenesis^13–17^.

Gram-negative bacteria *H. pylori*, which colonize on average 50% of the human population induce local inflammation in the stomach, development of gastric or duodenal ulcers, and even gastric cancer^18,19^. The majority of CHD patients are seropositive for anti-*H. pylori* antibodies indicating the exposure to the pathogen^20–24^. *H. pylori* produce many virulence factors, adhesins, toxins and pro-inflammatory proteins, which induce a multi-step process in pathogenesis on the level of gastric epithelial barrier, and possible also systemically^25,26^. Several hypotheses have been proposed to explain the role of *H. pylori* in CHD^27^. Transmission of *H. pylori* components through damaged gastric barrier and an interaction of these components with vascular endothelial cells or immunocompetent cells driving a systemic inflammation is possible^28–30^.

The interactions of Lewis (Le) determinants of *H. pylori* lipopolysaccharide (LPS) with E or L selectins may facilitate a survival of these bacteria within endothelium^31,32^. Although only few studies showed the presence of viable *H. pylori* or genomic material of these bacteria (16S rRNA) in atherogenic plaques^21,33,34^, *H. pylori* induced gastritis has been suggested as possible link in the development of coronary plaque^35^.

*Helicobacter pylori* isolates producing a cytotoxin associated gene A (CagA) protein are considered as more proinflammatory and due to this proatherogenic than non-CagA strains^36–38^. Also *H. pylori* heat shock protein (Hsp) B may drive vascular inflammatory response^31,32,39^.

The autoimmunity concept in the development of CHD has been suggested. This hypothesis is based on *H. pylori* driven antibodies cross-reacting with the host components including LeX/Y determinants, Hsp60 or tumor necrosis factor alpha receptor^23,39–41^.

In *H. pylori* infected vs non-infected CHD patients the serum levels of LDL, CRP, homocysteine, TNF-alpha and metalloproteinases (MMPs) are increased^15,28,42,43^, whereas serum paraxonase-1 is diminished in correlation with higher carotid-intima media thickness^44^. Homocysteine in excess damages blood vessels, and due to this a deposition of cholesterol in the vessels is facilitated. *H. pylori* by stimulation secretion of TNF-alpha by macrophages increases the serum level of triglicerides, and decreases the level of high density lipoprotein (HDL). The association between *H. pylori* infection and insulin resistance, which significantly increases the risk of CHD has been also suggested^45^.

Recently, Krupa et al., using *Caviae porcellus* model showed that *H. pylori* infection acts synergistically with high fat diet in development of proatherogenic endothelial cell environment^29^, and an increased arterial stiffness in *H. pylori* infected animals in conjunction with infiltration of inflammatory cells into the internal wall of the endothelium. A transformation of human monocytes or macrophages into foam cells in the milieu of *H. pylori* components in vitro has been also reported^29^. The concept that *H. pylori* infection may be involved in the development of CHD needs further elucidation. Particularly, finding the biomarkers differentiating specifically CHD patients infected with *H. pylori* is necessary.

Fourier-Transform Infrared Spectroscopy (FTIR) is based on measurement of the interaction of infrared radiation (IR) with matter by absorption, emission, or reflection and enables an identification of chemical substances or functional groups qualitatively and quantitatively. The transmission spectroscopy measures the intensity of the radiation passing through the sample, while the reflection spectroscopy measures the infrared radiation IR intensity, which is reflected from the sample under test^46^. IR spectroscopy has been used to qualitative and quantitative diagnostic of biological fluids like blood, serum, saliva and urine^47,48^.

The FTIR spectrum of human serum can be divided into groups of components with characteristic absorption bands: fatty acids (W1 3000–2800 cm^−1^), peptides and proteins (W2 1800–1500 cm^−1^), proteins, phosphate-carrying compounds and fatty acids (W3 1500–1200 cm^−1^), carbohydrates (W4 1200–900 cm^−1^). The part W5 (900–750 cm^−1^) corresponds to specific peaks unique for the sample^49,50^*.*

FTIR is also a powerful analytical technique used in pharmaceutical sciences or for evaluation of plasma biomarkers^51^, and for monitoring cellular alterations, including identification of leukemia and cancer cells^51–54^. FTIR was also used in the diagnostic of rheumatoid arthritis (RA)^55^***.*** Mordehai et al., revealed that FTIR microspectroscopy and cluster analysis have a potential to diagnose various infections^56^. Using FTIR it was possible to identify urinary tract pathogens^57^. We used FITR for monitoring experimental *H. pylori* infection and related inflammatory response in *Caviae porcellus* model^58^. We also applied this technique to analyze of selected soluble biomarkers, correlated with *H. pylori* infection in children and presumable consequent delayed growth^59^.

Recently, FTIR analysis of serum samples has been suggested as a promising method to diagnose the prevalence of atherosclerosis and its clinical manifestation prior to more advanced imaging procedures^60^. Marzec et al., using FTIR investigated the distribution and classes of atherosclerosis lesions in the vascular wall^61^. Micro-attenuated total reflection (ATR)-FTIR imaging has been applied ex vivo to investigate the contribution of inducible nitric oxide synthase to lesion composition in ApoE−/− mice^62^, as well as for selective detection of CRP^63^. FTIR was used for analysis of triglicerides, LDL structural diversity or protein structure^64–66^. Searching for biomarkers linking *H. pylori* infection and CHD is important for therapeutic reasons^24^. In our previous studies conducted in vitro on gastric epithelial cells and vascular endothelial cells we showed that *H. pylori* components may be involved in the elevation of proatherogenic inflammatory response corelated with induction of oxidative stress, which may provide environment for oxidation of LDL a classic risk factor of CHD^30^. *H. pylori* also upregulates the level of homocysteine, which in excess is pro-atherogenic^4^. Furthermore, *H. pylori* infection in *Caviae porcellus* fed with high fat diet increased the risk of development of endothelial pro-inflammatory milieu^29^. We expect that FTIR analysis of serum samples potentially may help to select a differentiating biomarkers with diagnostic value. In the current study we analyzed IR spectra of human sera from healthy donors, non-infected or infected with *H. pylori*, and from CHD patients infected with theses pathogenic bacteria to see whether FTIR is able to illustrate the differences in infrared spectra characteristic of proteins, lipids and carbohydrates. The IR spectra of serum samples means the absorption bands—windows (W) in the range of wavenumbers (3000–1050 cm^−1^) characteristic for biological samples. The biological markers that might be associated with the inflammatory response related to infection with *H. pylori* and CHD considered in this study were as follows: tumor necrosis factor (TNF)-α as one of the major inflammatory cytokines, C-reactive protein (CRP) as a marker of inflammation and homocysteine as potentially pro-inflammatory and pro-atherogenic factor, low density lipoprotein (LDL) and triglycerides as classic risk factors of CHD^7–12^.

## Results and discussion

*Helicobacter pylori* infection is characterized by ongoing chronic inflammation, determinants of which are: CRP, TNF-α, and homocysteine. In CHD patients CRP is increased as well as triglicerides and LDL, which are a classic CHD risk factors***.*** Homocysteine in excess is also pro-atherogenic. The purpose of this study was to determine the specific motives of IR spectra of serum samples correlating with *H. pylori* infection and mathematical models identifying sera of infected individuals. Furthermore, the best predictors related to CHD in *H. pylori* infected patients were under the study.

### *H. pylori* status

Out of 53 sera from healthy donors, 32 did not contain antibodies towards *H. pylori* glycine acid extract (GE) containing surface antigens of these bacteria. These subjects were defined as truly *H. pylori* negative. Anti-GE antibodies were detected in 21 healthy donors, indicating that they were exposed to *H. pylori* infection, although they remained asymptomatic. By comparison, all 90 sera of CHD patients were positive for anti-GE IgG (Table 1).

**Table 1:** The level of IgG anti-*H. pylori* GE in human serum.

Study groups	N	*H. pylori* negative without IgG anti-*H. pylori*	*H. pylori* positive with IgG anti-*H. pylori*
Healthy donors	53	32 (OD_450_ = 0.254 ± 0.069)	21 (OD_450_ = 1.610 ± 0.580)
CHD patients	90	0	90 (OD_450_ = 1.847 ± 0.610)

*Cut off* OD_450_ = 0.67 ± 0.049; *GE* glycine extract from the reference *H. pylori* strain, *CHD* coronary heart disease.

The correlation between anti-*H. pylori* antibodies induced during infection with these bacteria, potentially cross reacting with components of vascular endothelium, and development of CHD has been suggested^39–41^. In CHD patients infected with *H. pylori* some components of these bacteria may induce antibodies, potentially autoantibodies, which may cross-react with components of vascular endothelium e.g. Hsp60 or TNF-alpha receptor (TNFR)^39–41^, which are elevated in atherogenic endothelium. The potential mechanism of increasing of inflammatory response in such milieu might depend on complement related cytotoxicity or blocking functional cell receptors.

In this study the level of anti-*H. pylori* antibodies in CHD patients was found higher than in non CHD *H. pylori* positive healthy donors. Previously we showed that, the level of such antibodies in CHD patients was even higher than in *H. pylori* positive patients with gastritis^23^. It could be due to domination of a T helper 2 lymphocyte response in CHD patients, which promotes the antibody production^67^. It is also possible that prophylactic usage by CHD patients of acetylsalicylic acid (ASA) as antiaggregating factor, due to its’ local cytotoxicity, can increase the penetration of *H. pylori* antigens through epithelial barrier and increase their systemic availability to immunocompetent cells^30^.

Up to now the relationship between *H. pylori*-induced gastritis and atherosclerosis, and CHD remains unclear. Among other entities, *H. pylori* has been implicated in the pathogenesis of the metabolic syndrome with an arterial hypertension, which is a risk factor for cardio-cerebrovascular diseases^1^.

### Analysis of IR spectra of human sera

**Figure 1:**
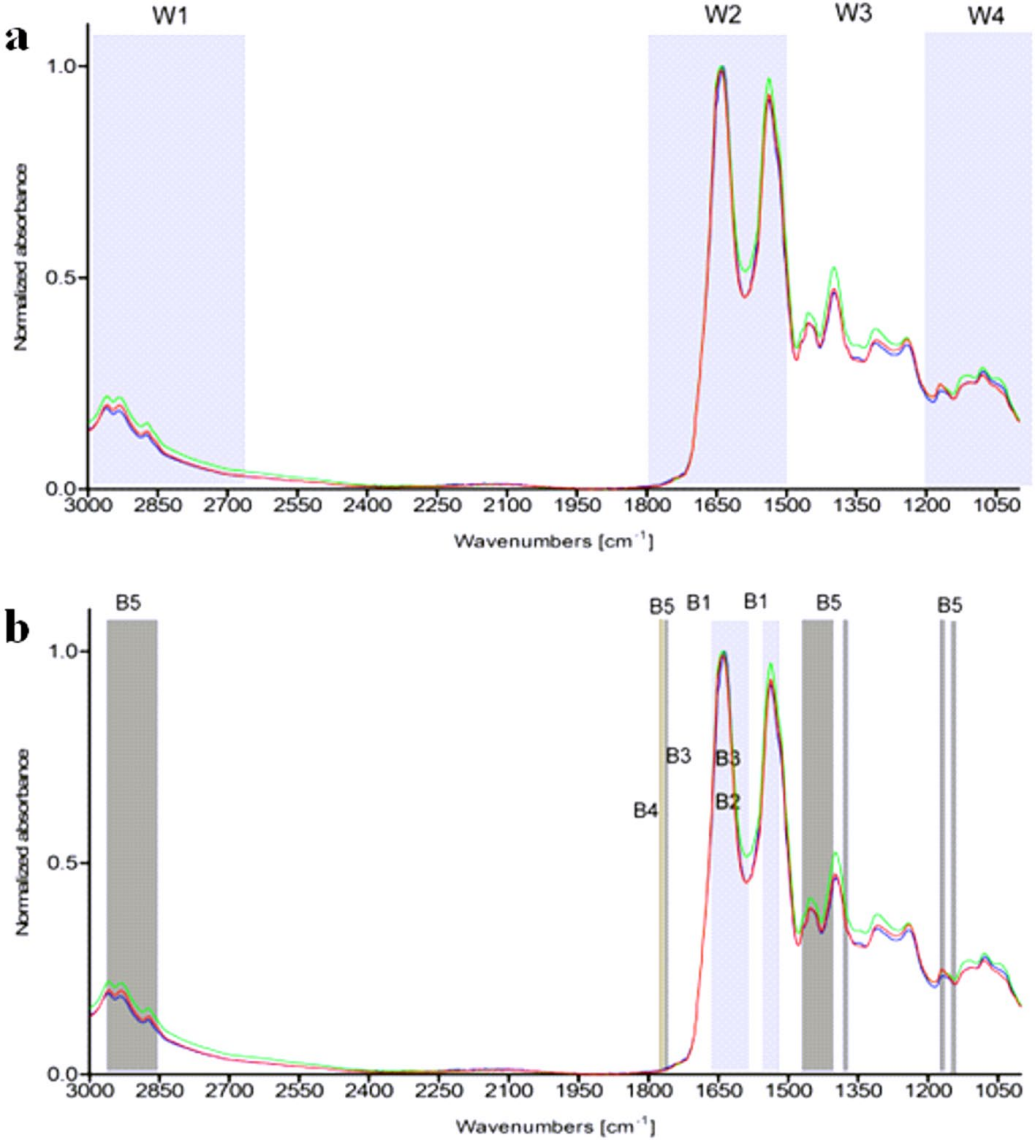
Representative infrared spectra of investigated human sera: green—CHD patients infected with *H. pylori*, red—healthy blood donors *H. pylori* positive and blue—*H. pylori* negative healthy individuals. (**a** representative spectra. W1–W4 correspond to the characteristic fragments of fatty acids (W1 3000–2800 cm^−1^); peptides and proteins (W2 1800–1500 cm^−1^); proteins, phosphate-carrying compounds and fatty acids (W3 1500–1200 cm^−1^); carbohydrates (W4 1200–900 cm^−1^). (**b**) Part of IR spectra most differentiating the study groups. B1–B5 show characteristic absorption band differentiating in this study, see Table 2.

Several groups of components with characteristic absorption bands can be selected in the IR spectrum of serum: fatty acids (W1 3000–2800 cm^−1^), peptides and proteins (W2 1800–1500 cm^−1^, proteins, phosphate carrying compounds and fatty acids (W3 1500–1200 cm^−1^), carbohydrates (W4 1200–900 cm^−1^) (Fig. 1a). Vibration band assignment has been carried out on the infrared spectrum of serum by comparing the position, relative intensity and shape of the bands with the bands of related molecules. Considerable differences in infrared spectra characteristic from lipids, proteins as well as carbohydrates have been observed in tested serum samples from: healthy blood donors seronegative or seropositive for anti-*H. pylori* IgG, and patients with CHD infected with *H. pylori*. Representative spectra of sera for wavelengths 3000–1000 cm^−1^ are presented in Fig. 1a. We demonstrated differences in the absorption spectra for selected biomolecules: triglicerides, CRP, homocysteine, LDL and TNF-α between CHD patients infected with *H. pylori* and healthy blood donors seronegative or seropositive for anti-*H. pylori* Table 2, Fig. 1a,b. Detailed analysis of infrared spectra allowed the theoretical adjustment of the individual peak to the corresponding bonds and chemical rubble as well as to the relevant biomolecules (Fig. 1a,b, Table 2). A vibration band assignment was done to show frequencies of the chemical groups in the tested sample. The spectral region (B1) corresponds to CRP. This region contains the following prominent absorption peaks: 1545 cm^−1^ is due to N–H in plane bending vibration strongly coupled to C–N stretching vibration protein (amide I); 1632–1652 cm^−1^ represents response to NH_2_ scissoring (amide I) (Fig. 1a,b, Table 2). The absorption band (B2) (amide I and amide II) is characteristic for homocysteine vibration peak: 1600–1550 cm^−1^, NH_2_ scissoring and N–H in plane bending vibration strongly coupled to C–N stretching vibration protein (Fig. 1a,b, Table 2). The spectral region (B3) corresponds to the vibration band characteristics of LDL and typical region for peptides and proteins (amide I) (Table 2 and Fig. 1a,b). In this spectrum, C=O stretching, N–H, CO_2_ asymmetric stretching (1740–1720 cm^−1^), C=O symmetric stretching and NH_2_ scissoring (1660–1600 cm^−1^ 1650 cm^−1^) vibrations are present (Fig. 1a,b, Table 2). The prominent absorption peak 1760–1740 cm^−1^ (B4) was identified for TNF-α due to the C=O stretching (amide I) of proteins (Fig. 1a,b, Table 2). The absorption bands response to triglycerides (B5) at 2925–2850 cm^−1^ correspond to methylene asymmetric stretching (lipids), 1740 cm^−1^ is attributed to amide I–C–H symmetric stretching of CH_2_ group, 1475–1470 cm^−1^ is attributed to methylene scissoring, 1460–1440 cm^−1^ and 1385 cm^−1^ are attributed to methyl asymmetric deformation and 1180 cm^−1^ as well as 1115 cm^−1^ are C–O symmetric vibrations corresponding to carbohydrates (Fig. 1a,b, Table 1).

**Table 2:** Identified reference molecules included in the composition of IR spectra of tested human sera.

Molecule	Absorpcion band (cm^−1^)	ID	Description	Component group	References
CRP	1545	[B1]	N–H in plane bending vibration strongly coupled to C–N stretching vibration protein	Amide II	^63^
	1632–1652		NH_2_ scissoring	Amide I	
Homocysteine	1600–1550	[B2]	NH_2_ scissoring	Amide I	^12^
			N–H in plane bending vibration strongly coupled to C–N stretching vibration protein	Amide II	
LDL	1740–1720	[B3]	C=O stretching	Amide I	^66^
	1660–1600		C=O symmetric stretching	Amide I	
			NH_2_ scissoring	Amide I	
TNF-α	1650	[B4]	C=O stretching	Amide I	^68^
	1760–1740				
Triglycerides	2925–2850	[B5]	Methylene asymmetric stretching	Lipids	^51^
	1740		C=O stretching	Amide I	
	1475–1470		Methylene scissoring		
	1460–1440		Methyl asymmetric deformation	Amino acid	
	1385		Methyl symmetric deformation	Amino acid	
	1180		C–O symmetric stretching	Carbohydrates	
	1115		C–O symmetric stretching	Carbohydrates	

ID symbol shows representative characteristic fragment of infrared spectrum, of human serum, for the molecules differentiating the study groups presented on Fig. 1a,b: patients with coronary heart disease (CHD) infected with *H. pylori*, healthy donors non-infected or infected with *H. pylori*. *B* band, *CRP* C-reactive protein, *LDL* low density lipoprotein, *TNF-α* tumor necrosis factor alpha.

### Wavenumbers correlating with *H. pylori* infection and mathematical models identifying sera of infected individuals

The principles and results of the differentiation of infected and non-infected individuals are presented in the Tables 3, 4 and 5. In window W3 the highest frequency of wave numbers was shown, which differentiate individuals infected with *H. pylori* from uninfected. The molecule that most likely differentiates them are triglycerides (band B5) (Fig. 1a,b). The increase in absorbance maximum, which can also differentiate groups, is visible in the window: W1 (absorbance band 2954 cm^−1^, band B5-lipids/triglycerides, W2 (absorbance band 1545 cm^−1^, band B1—CPR), W3 (absorbance band 1255 cm^−1^), although the differences in peak heights are not statistically significant (Fig. 1a,b). It is expected that further studies using more samples will confirm this trend.

**Table 3:** Best predictors for *H. pylori* infection.

Window	Absorption band (cm^−1^)	χ^2^-square test value	*p*-value	Teoretical chemical bond
W3	1425	66.67	3.30E−16	C=O stretching
	1354	62.44	2.80E−15	C–N stretching
	1353	62.44	2.80E−15	C–H bending
	1424	61.21	5.10E−15	C=O stretching
	1355	59.40	1.30E−14	C–H bending

**Table 4:** Discrimination of serum samples from *H. pylori* seropositive vs seronegative individuals based on IR spectra and artificial neural networks.

No	Topology	Correct classification percentage		Error function	Activation function	
					Hidden neurons	Output neurons
		Trening subset	Validation subset			
1	MLP 5-5-2	98.73	96.15	Entropy	Tanh	Softmax
2	MLP 5-4-2	94.94	89.74	Entropy	Tanh	Softmax
3	MLP 4-4-2	94.94	89.74	Entropy	Tanh	Softmax
4	MLP 4-5-2	96.20	89.74	Entropy	Exponential	Softmax
5	MLP 5-3-2	94.94	88.46	Entropy	Tanh	Softmax
6	MLP 5-2-2	97.47	88.46	Entropy	Tanh	Softmax
7	MLP 4-3-2	93.67	88.46	Entropy	Tanh	Softmax
8	MLP 3-5-2	94.94	88.46	Entropy	Logistic	Softmax
9	MLP 3-4-2	94.94	88.46	SOS	Logistic	Identity
10	MLP 2-4-2	93.67	85.90	SOS	Logistic	Identity

**Table 5:** Quality indicators of ANN No. 1. The indicators were calculated based on the results from the validation subset.

	Name of measure	Value
1	Total number of sampels	78
2	True positive (TP)	52
3	False positive (FP)	2
4	False negative (FN)	1
5	True negative (TN)	23
6	True positive rate (TPR)	0.9811
7	False negative rate (FNR)	0.0189
8	True negative rate (TNR)	0.92
9	False positive rate (FPR)	0.08
10	Positive predictive value (PPV)	0.963
11	False discovery rate (FDR)	0.037
12	False omission rate (FOR)	0.0417
13	Negative predictive value (NPV)	0.9583
14	Positive likelihood ratio (LR+)	12.2642
15	Negative likelihood ratio (LR−)	0.0205
16	Diagnostic odds ratio (DOR)	598
17	Accuracy (ACC)	0.9615
18	Informedness	0.9011
19	Markedness	0.9213

Based on the chi-square test, 5 predictors (wavenumbers) were chosen for ANNs design—Table 3. All these wavenumbers belong to window W3 (Fig. 1a,b). This part of IR spectrum is called “mix region”, because many chemical compounds (i.e. proteins, P-carrying chemical compounds) play important role in this window. The location of all 5 wavenumbers in the range of W3 indicate that different chemical compounds are related to *H. pylori* infection.

Many neural networks have been created that differ in the number of neurons in the layers (2–5), activation functions (linear, logistic, hyperbolic tangent, exponential, sine) and error function (sum of squares, entropy). Discrimination of serum samples from *H. pylori* seropositive vs seronegative individuals based on IR spectra and artificial neural networks is showed in Table 4. The best results were obtained using artificial neural network with 5 input neurons and 5 hidden neurons. This ANN is characterized by high accuracy of 96.15%—Table 5. The increasing complexity of the network (measured by the number of connections between neurons) improves the accuracy of the models.

The quality of ANNs was assessed by 13 indicators based on the number of correctly and incorrectly classified cases. The best ANN is characterized by a high true positive rate (TNR) and true negative rate (TNR) of 98% and 92%, respectively. The value of the diagnostic odds ratio (DOR) is 598, so the neural network discriminates correctly the serum samples.

### Discrimination between serum samples from CHD patients seropositive for anti-*H. pylori* antibodies vs *H. pylori* seronegative healthy individuals

Based on the chi-square test, 4 predictors (wavenumbers) were chosen for ANNs design (Table 6). The wavenumbers belong to window W3, and one belongs to window W2. This part of IR spectrum is called “mix region”, because many chemical compounds (i.e. proteins, peptides P-carrying chemical compounds) play important role in this window. The location of all 4 wavenumbers in the range of W3 and W2 indicate that different chemical compounds are related to *H. pylori* infection, and absorption maxima are increased in CHD patients infected with *H. pylori* in relation to noninfected individuals (Fig. 1).

**Table 6:** Best predictors for differentiation of CHD patients infected with *H. pylori*.

Window	Absorpcion band (cm^−)^]	χ^2^-square test value	*p*-value	Teoretical chemical bond
W3	1425	59.66	2.22 × 10^–14^	C=O stretching
	1353	50.04	1.03 × 10^–8^	C–H bending
	1424	58.11	3.15 × 10^–10^	C=O stretching
W2	1545	59.76	2.33 × 10^–14^	C–N stretching

The W2 and W3 windows showed the highest frequency of wave numbers, which differentiates CHD patients infected with *H. pylori* from healthy uninfected individuals (Table 6). The absorbance maxima significantly differentiating between these two groups were found in the W3 window, and the molecules that most likely differentiates them are triglycerides (band B5, Fig. 1a,b). In addition, in the W2 window, one maximum absorbance is characteristic of CRP (band B1, Fig. 1a,b) and significantly differentiates the tested groups. In general, the higher triglyceride level, the higher cardiovascular risk. Reduction of cardiovascular events with Icosapent Ethyl-Intervention Trial (REDUCE-IT) study confirmed that the risk of major ischemic events, was significantly lower in covalent ethyl treated patients with elevated triglyceride levels confirming the correlation between the higher triglyceride level and CHD^69,70^. It has been also revealed that there is a link between CRP level and coronary events, however it is rather due to the intensity of inflammatory response, which drives the CRP production than the association of a genetic variant (rs1130864), which is known to be related to CRP levels^11,71^.

Wave numbers for which absorbance maxima are also higher in patients with CHD exposed to *H. pylori* compared to uninfected healthy individuals are visible in the window W2 (absorbance band 1395 cm^−1^ and 1350 cm^−1^). Differences in peak heights are statistically significant, however, based on literature data, it was not possible to assign a specific molecule to these bands. Further studies are needed to specify these particles, which will significantly increase the ability to differentiate *H. pylori*-infected CHD patients.

We also checked whether the biomarkers, which were increased in healthy donors *H. pylori* positive were further upregulated in CHD patients infected with these bacteria. It was revealed that the maximum absorption rate for CRP (B1, absorbance band 1545 cm^−1^) and triglycerides (B5, absorbance band: 2955 cm^−1^, 2850 cm^−1^ and 1412 cm^−1^) were increased in CHD patients infected with *H. pylori* vs healthy donors *H. pylori* positive.

The maximum absorption rate for CRP (B1, absorbance band 1545 cm^−1^) and triglycerides (B5, absorbance band: 2955 cm^−1^, 2850 cm^−1^ and 1412 cm^−1^) was increased in CHD patients *H. pylori* positive as compared to healthy donors *H. pylori* positive. In addition, in the analyzed IR spectrum at the following wavelength ranges: 3000–2400 cm^−1^, 1600–1560 cm^−1^, and 1500–1050 cm^−1^, the maximum absorbance in *H. pylori* infected CHD patients was higher than in healthy *H. pylori* positive or *H. pylori* negative individuals (Fig. 1).

The best results differentiating CHD patients infected with *H. pylori* and healthy donors *H. pylori* negative were obtained using artificial neural network with 4 input neurons and 2 hidden neurons (Table 7). This ANNs is characterized by high accuracy of 95.11% (Table 8). The increasing complexity of the network (measured by the number of connections between neurons) improves the accuracy of the model.

**Table 7:** Best predictors for differentiation CHD patients infected with *H. pylori* from *H. pylori* negative healthy donors based on IR spectra and artificial neural networks.

No	Topology		Correct classifications percentage		Error function	Activation function	
						Hidden neurons	Output neurons
			Trening subset	Valiadation subset			
1	MLP 4-2-2		93.98	93.58	SOS	Logistic	Identity
2	MLP 4-3-2		93.65	92.30	SOS	Logistic	Identity
3	MLP 4-2-2		93.55	92.30	SOS	Logistic	Identity
4	MLP 2-4-2		89.87	89.74	Entropy	Tanh	Softmax
5	MLP 2-5-2		89.87	89.74	Entropy	Exponential	Softmax
6	MLP 3-3-2		91.139	87.17	SOS	Tanh	Tanh
7	MLP 3-5-2		91.139	85.89	Entropy	Tanh	Softmax
8	MLP 3-2-2		89.873	85.89	Entropy	Tanh	Softmax
9	MLP 3-4-2		89.873	84.615	Entropy	Tanh	Softmax

**Table 8:** Quality indicators of ANN No. 1. The indicators were calculated based on the results from the validation subset.

	Name of measure	Value
1	Total number of sampels	111
2	True positive (TP)	88
3	False positive (FP)	4
4	False negative (FN)	0
5	True negative (TN)	19
6	True positive rate (TPR)	0.9715
7	False negative rate (FNR)	0.0285
8	True negative rate (TNR)	0.91
9	False positive rate (FPR)	0.09
10	Positive predictive value (PPV)	0.961
11	False discovery rate (FDR)	0.039
12	False omission rate (FOR)	0.0317
13	Negative predictive value (NPV)	0.9581
14	Positive likelihood ratio (LR+)	12.2631
15	Negative likelihood ratio (LR−)	0.0202
16	Diagnostic odds ratio (DOR)	597
17	Accuracy (ACC)	0.9511
18	Informedness	0.9021
19	Markedness	0.9211

The quality of ANNs was assessed by 13 indicators based on the number of correctly and incorrectly classified cases. The best ANN is characterized by a high true positive rate (TPR) and true negative rate (TNR) of 97% and 91%, respectively. The value of the diagnostic odds ratio (DOR) is 597, so the neural network discriminates correctly the serum samples.

*Helicobacter pylori* is one of the most common human pathogens, which colonize gastric mucosa. Gastritis due to *H. pylori* infection is associated with an increased level of CRP and homocysteine due to decreased absorption of vitamin B12 and folic acid. Thus *H. pylori* infection and related gastritis in conjunction with an increased CRP and homocysteine levels may be the earliest events in the process of atherosclerosis and plaque formation^35^. Vahdat et al. revealed that beyond traditional cardiovascular risk factors, concomitant chronic infection, including *C. pneumoniae*, *Herpes simplex virus* type 1 (HSV-1), *H. pylori* and cytomegalovirus (CMV), and elevated CRP are significantly correlated with electrocardiogram-defined CHD^42^. Our FTIR results remain in agreement with the above data indicating triglicerides and CRP as potential biomarkers linking *H. pylori* infection accompanied by gastritis with the development of CHD. However, besides *H. pylori* infection, the metabolic status of the patient may be important or interaction between the two. As we showed previously on *Caviae porcellus* model the high fat diet might determine the lipid related metabolic status^29^. The missing link in this study is the lack of *H. pylori* negative patients with CHD, which might be an important factor influencing the results. Further study with CHD patients non-infected with *H. pylori* will let us answer the above doubt and confirm the conclusions derived from the current preliminary study.

## Conclusion

Obtained results indicate that IR spectroscopy in combination with ANNs can be helpful tool for monitoring of *H. pylori* infected patients with CHD. Detection of characteristic serum profile in CHD patients infected with *H. pylori* may possess an early diagnostic value. It is expected that high quality mathematical models can be used in a future as tools for rapid bacterial detection in this group.

## Materials and methods

### Patients and controls

Informed consent was obtained from all subjects. This study was approved by the Bioethical Committee in the Medical University in Lodz (protocol: RNN/134/13/KE/2-13; 34/KBBN-UŁ/I/201 and 10/KBBN-UŁ/II/2015). All experiments were performed in accordance with the approved guidelines.

The first study group consisted of patients with CHD. These subjects were under the care of the 2nd Cardiology Clinic of Bieganski Regional Hospital (Medical University of Lodz, Poland) and the Department of Internal Diseases and Clinical Pharmacology (Medical University of Lodz, Poland) in whom CHD disease was confirmed by coronary radiography, physiological parameters and biochemical tests. The CHD group consisted of 90 patients of both sexes. The inclusion criteria for the CHD group included classic CHD risk factors and were as follows: arterial hypertension (intake of antihypertensive drugs prior to enrolment, systolic blood pressure > 140 or diastolic blood pressure > 90 mmHg), diabetes (glucose-reducing agents or insulin intake prior to enrolment), fasting glucose level (> 126 mg/dL (7 mmol/L) double checked), glycosylated hemoglobin (Hb) level (> 7% or positive oral glucose tolerance test with 75 g glucose), dyslipidemia (hypolipidemic agents intake prior to enrolment), hyperlipidemia: total cholesterol level [> 190 mg/dL (5.0 mmol/L), LDL cholesterol level (> 115 mg/dL (3.0 mmol/L)], high-density lipoprotein (HDL) cholesterol [< 40 mg/dL (1.0 mmol/dL)], or triglycerides [> 150 mg/dL (1.2 mmol/L)], obesity measured by the body mass index (BMI) (> 32), nicotinism. Demographic and clinical characteristic of the CHD group was as follows: age range 38–81 (mean age 59.7 ± 7.4, males 73.9%), arterial hypertension 59.7%, systolic/diastolic blood pressure range 100–195/60–115 (mean 130 ± 21/mean 79 ± 11, diabetes mellitus type 1/2 0/22.8%, smoking 40.2%, obesity 13.0%, BMI range 19.1–37.4 (mean 27.2 ± 3.9), gastric/duodenal ulcer disease 6.4%, dyslipidemia 59.7%, total cholesterol range 37–347 (mean 205 ± 49), LDL range 38–243 (mean 132 ± 48), HDL range 19–80 (mean 43 ± 11), triglicerides range 37–478 (mean 148 ± 77), history of myocardial infarction (85.8%), history of stroke (6.38%), infarction (85.8%), primary coronary angioplasty (68.5%). The second study group consisted of healthy donors—control group, (53 individuals of both sexes, mean age 56.5 ± 5.2, males 65.9%), with a negative history of cardiovascular and gastric/duodenal ulcer diseases. These subjects have been recruited from one of the basic health care units in Lodz, Poland, and were selected on the basis of the exclusion of classic CHD risk factors: arterial hypertension, hyperlipidemia, diabetes, smoking, obesity as well as dyspepsia, abdominal pain or gastric bleeding. The *H. pylori* status in healthy donors and some CHD patients was estimated by the ^13^C urea breath testing (^13^C UBT)^72^, and the enzyme linked immunosorbent assay (ELISA) for IgG antibodies against the *H. pylori* antigenic complex called a glycine extract (GE)^73^. This laboratory ELISA was standardized using the reference serum samples from *H. pylori* positive or *H. pylori* negative subjects diagnosed according to the “gold standard”, including culture of *H. pylori* from the biopsy specimens, rapid urease test, and histological examination for Helicobacter-like organisms, as well as immunoblot for anti-*H. pylori* IgG (MileniaBlot *H. pylori*, DPC Biermann, GmbH, Bad Nanheim, Germany)^73^.

### ELISA for anti-*H. pylori* IgG

The ELISA assay for anti-*H. pylori* IgG was performed using *H. pylori* antigenic complex—glycine acid extract (GE), which was obtained by extraction of surface antigens from the reference *H. pylori* strain CCUG (Culture Collection University of Gothenburg, Sweden) 17874, CagA and vacuolating cytotoxin A (VacA) positive, with glycine acid buffer as previously described^73^. Major proteins in GE recognized by the reference sera from *H. pylori* infected patients were as follows: 120 kDa (CagA), 87 kDa (VacA), 66 kDa (urease B, UreB), 60 kDa (Hsp), 29 kDa (urease A, UreA), between 66 and 22 kDa^73^. The GE protein concentration was 600 μg/mL (NanoDrop 2000c Spectrophotometer, Thermo Fisher Scientific, Waltham Massachusetts, USA). The GE contained < 0.001 EU/mL of LPS, as shown by the chromogenic *Limulus amebocyte* lysate test (Lonza, Braine-Alleud, Belgium).

### The measurement of infrared spectra and its processing

IR spectra measuring of human sera as previously described^27,28,43^. IR spectra of 141 human sera (90 sera of CHD and 53 from healthy donors) were evaluated by the ATR-FTIR technique using a FT-IR/FT-NIR Spectrum 400 spectroscope (PerkinElmer, Waltham, Massachusetts, USA). The spectrometer was equipped with an ATR adapter with a single reflection diamond crystal. Blood samples for serum preparation, were collected from each tested individual in 4 various test tubes. One IR spectrum of serum was measured from each tube. For each sample, 25 scans were performed. A total number of 564 IR spectra were measured: learning subset (423 cases) and validating subset (141 cases).

Serum samples were stored at − 80 °C until the measurement was made. Sera were thawed at 20 °C using a CH-100 Bioscan (Bionovo, Legnica, Poland) thermo block and then shaken for 30 s by using Lab Dancer vario (IKA, Shanghai, China). Measurements were performed at 20 °C, at constant air humidity. Before each measurement, the spectroscope crystal was cleaned with 95% alcohol and a baseline measurement was made. One microliter of serum was added at the spectroscope crystal and left for water evaporation. The amount of evaporated water was estimated from the intensity of a band 3400–3200 cm^−1^. It was assumed that the water was evaporated from the sample when the band intensity around the wavenumber of 3300 cm^−1^ decreased to about 40% of the original value. The process of water evaporation in these conditions lasted about 5 min. The spectrum of the sediment remaining on the crystal was measured in the range of wavenumbers 4000–650 cm^−1^ with a resolution of 1 cm^−1^ and then preprocessed in two steps: (a) calculation of the first derivative by five-point stencil, (b) normalization to the range {0.1}. The first derivatives were used for mathematical analysis^55,58,59^. The biological markers that are associated with the inflammatory reaction following infection with *H. pylori* and CHD were then assessed in the obtained human serum IR spectrum. We were searching for TNF-α being major pro-inflammatory cytokine, CRP and homocysteine upregulated during infections, including an infection with *H. pylori*, and tissue damage, LDL and triglycerides, which are classic risk factors of CHD^7–12^.

### Mathematical model developed for patients’ differentiation

In this study the perceptron model was used in designing artificial neural networks (ANNs)^75–77^. The perceptron's task is to classify the vector x given as input into one of the two classes, C_1_ or C_2_. If the output signal of the neuron takes the value of 1, the vector x is classified to class C_1_, if it takes the value of 0, then to class C_2_. The perceptron divides the N-dimensional space of input vectors into two half-spaces separated by an N^−1^-dimensional hyperplane. This hyperplane is called the decision boundary. If the space of objects is two-dimensional, then the border separating them is a straight line.

Learning the perceptron is about finding its weight values. The difference between the expected value of the neuron's output and the obtained value is the error made by the neuron when presenting the j-th example:

$$\delta j=dj-yj.$$

Our goal was to find a solution that minimizes the following perceptron criterion function

$$J\left(w\sim \right)=\sum_{Xi,di}wTx+w0(-w\sim tx-w\sim 0).$$

In the above criterion function, summing was done over incorrectly classified objects, which leads to minimizing the number of incorrect classifications. In practice, an equivalent notation is often used

$$J\left(w\sim \right)=\sum_{Xi,di}wTx+w0(-w\sim tx),$$

where x = [1, × 1, × 2, …, × N] T. The search for the minimum of the perceptron criterion function is equivalent to minimizing the mean square error.

$$Q=12\sum j=1N{(dj-yj)}^{2}=\sum j=1NQ2j,Qj=12\delta 2j,$$

where δ is given by the formula. The minimum of the above criterion function is searched for by the method of the highest gradient decrease^74–76^.

Starting from the random solution in each learning step, to the current weight values, the correction was added directly proportionally to the gradient of the criterion function at the point w (current weight value) according to the formula:

$$wk+1=wk-\upeta \Delta \left(\mathrm{J}\left(\mathrm{wk}\right)\right).$$

The chi-square statistical test was used to check the part of the IR spectra, which correlated with the examined feature. Next, a number of mathematical models based on multilayer perceptrons (a type of ANN) were built.

The best predictors (wavenumbers) were used as input data for ANNs: 5 wavenumbers differentiate the tested samples for *H. pylori* infection (*H. pylori* infected individuals vs healthy individuals noninfected); 4 wavenumbers differentiate the tested samples for CHD *H. pylori* infected patients (CHD *H. pylori* infected individuals vs *H. pylori* noninfected individuals); All tested ANNs have similar topology: 2–5 input neurons, 1–5 hidden neurons and 2 output neurons.

The quality of the network was evaluated on the basis of the number of correct classifications in the validation set. The calculations were carried out using Statistica 13 software (StatSoft, Round Rock, TX, USA). More complex ANNs were not tested due to log time of necessary computing. All spectra were divided into two separate groups: learning set and validating set. The quality of the ANNs was evaluated on the basis of calculated number of correct classifications in the validating set in terms of sensitivity, miss rate, specificity, false positive rate, precision, false discovery rate, false omission rate, negative predictive value, positive likelihood ratio, negative likelihood ratio, accuracy, informedness, and markedness.

Selected ANAs were validated in terms of: false negative (FN), true positive (TP), informedness, markedness, condition positive (P)—number of real positive cases in the data, condition negative (N)—number of real negative cases in the data according to the following formulas:

- sensitivity as well as true positive rate (TPR): TTR = $\frac{TP}{P}$= $\frac{TP}{TP+FN}$ = 1 – FNR,
- specificity as well as true negative rate (TNR):
  $$\mathrm{TNR}\hspace{0.17em}=\hspace{0.17em}\frac{TN}{N}= \frac{TN}{TN+FP}=1-\mathrm{FPR},$$
- miss rate as well as false negative rate (FNR): $FNR\hspace{0.17em}=\hspace{0.17em}\frac{FN}{P}=\frac{FN}{FN+TP}=1-TPR,$
- false positive rate (FPR): $FPR\hspace{0.17em}=\hspace{0.17em}\frac{FP}{N}=\frac{FP}{FP+TN}=1-TNR,$
  were FP is a number of false positives, TN is the number of true negatives and N = FP + TN.
- precision (PPV): *PPV* = $\frac{TP }{TP+FP},$
- false omission rate (FOR): *FOR* = $\frac{FN}{FN+TN}=1-NPV,$
- negative predictive value (NPV): *NPV* = $\frac{TN}{TN+FN}=1-FOR,$
- false discovery rate (FDR): *FDR* = $\frac{FP}{FP+TP}=1-PPV,$
- positive likelihood ratio (PLR): *PLR* = $\frac{TPR}{100-TNR},$
- negative likelihood ratio (NLR)*: NLR* = $\frac{100-TPR}{TNR},$
- accuracy: *accuracy* = $$\frac{TN+TP}{TN+FP+TP+FN}.$$

### Statistical analysis

Statistical software STATISTICA version 13.3 software (https://statistica.software.informer.com/13.3/; StatSoft, Round Rock, TX, USA) was used for design and training the ANNs.

## Data Availability

All data generated or analyzed during this study are included in this published article.

## References

[1] Kountouras J (2021). Impact of *Helicobacter pylori*-related metabolic syndrome parameters on arterial hypertension. Microorganisms..

[2] Kowalski M (2001). Prevalence of *Helicobacter pylori* infection in coronary artery disease and effect of its eradication on coronary lumen reduction after percutaneous coronary angioplasty. Dig. Liver Dis..

[3] Moniyama Y, Adachi H, Fairweather D, Ishizaka N, Saita E (2014). Inflammation, atherosclerosis and coronary artery disease. Clin. Med. Insights Cardiol..

[4] Tamura T, Fujioka T, Nasu M (2002). Relation of *Helicobacter pylori* infection to plasma vitamin B12, folic acid, and homocysteine levels in patients who underwent diagnostic coronary arteriography. Am. J. Gastroenterol..

[5] Kutuk O, Basaga H (2003). Inflammation meets oxidation: NF-kappa B as a mediator of initial lesion development in atherosclerosis. Trends Mol. Med..

[6] Shishehbor MH, Bhatt DL (2004). Inflammation and atherosclerosis. Curr. Atheroscler. Rep..

[7] Calabro PE, Golia E, Yeh T (2009). CRP and the risk of atherosclerotic events. Semin. Immunopathol..

[8] Li H (2018). Inflammatory biomarkers of coronary heart disease. Front. Bios Scholar..

[9] Koenig W (2001). Inflammation and coronary heart disease: An overview. Cardiol. Rev..

[10] Libby P, Theroux P (2005). Pathophysiology of coronary artery disease. Circulation.

[11] Casas JP (2006). Insight into the nature of the CRP-coronary event association using Mendelian randomization. Int. J. Epidemiol..

[12] Bright ATS, Gunasekaran RRDS (2010). FTIR spectral analysis of plasma homocysteine levels among smokers. Asian J. Chem..

[13] Danesh JR, Peto CR (1997). Chronic infection and coronary heart disease: Is there any link?. Lancet.

[14] Saikku P (1998). Serological evidence of an association of a novel *Chlamydia,* TWAR, with chronic coronary heart disease and acute myocardial infarction. Lancet.

[15] Laurila A (1999). Association of *Helicobacter pylori* infection with elevated serum lipids. Atherosclerosis.

[16] Chmiela M (2003). A link between *Helicobacter pylori* and/or *Chlamydia* spp. infections and atherosclerosis. FEMS Immunol. Med. Microbiol..

[17] Bourgeois D, Inquimbert C, Ottolenghi L, Carrouel F (2019). Periodontal pathogens as risk factors of cardiovascular diseases, diabetes, rheumatoid arthritis, cancer, and chronic obstructive pulmonary disease—Is there cause for consideration?. Microorganisms..

[18] Clyne MB, Reeves DEP (2007). Bacterial factors that mediate colonization of the stomach and virulence of *Helicobacter pylori*. FEMS Microbiol. Lett..

[19] Chmiela M, Karwowska Z, Gonciarz W, Allushi B, Stączek P (2017). Host pathogen interactions in *Helicobacter pylori* related gastric cancer. World J. Gastroenterol..

[20] Mendall MA (1994). Northfield. Relation of *Helicobacter pylori* infection and coronary heart disease. Br. Heart J..

[21] Kowalski M (2002). Detection of *Helicobacter pylori* specific DNA in human atheromatous coronary arteries and its association to prior myocardial infarction and unstable angina. Dig. Liver Dis..

[22] Park MJ (2011). Association between *Helicobacter pylori* seropositivity and the coronary artery calcium score in a screening population. Gut Liver..

[23] Matusiak A (2016). Putative consequences of exposure to *Helicobacter pylori* infection in patients with coronary heart disease in terms of humoral immune response and inflammation. Arch. Med. Sci..

[24] Furuto Y (2021). Relationship between *Helicobacter pylori* infection and arteriosclerosis. Int. J. Gen. Med..

[25] Posselt G, Backert S, Wessler S (2013). The functional interplay of *Helicobacter pylori* factors with gastric epithelial cells induces a multi-step process in pathogenesis. Cell Commun. Signal..

[26] Chmiela M, Kupcinskas J (2019). Review: Pathogenesis of *Helicobacter pylori* infection. Helicobacter.

[27] Chmiela M, Gajewski A, Rudnicka K (2015). *Helicobacter pylori* vs coronary heart disease-searching for connections. World J. Cardiol..

[28] Tsay FW, Hsu PI (2018). *H. pylori* infection and extra-gastroduodenal diseases. J. Biomed. Sci..

[29] Krupa A (2021). *Helicobacter pylori* infection acts synergistically with high fat diet in a development of proinflammatory and potentially proatherogenic endothelial cell environment in an experimental model. Int. J. Mol. Sci..

[30] Gajewski A (2022). Accumulation of deleterious effects in gastric epithelial cells and vascular endothelial cells in vitro in the milieu of *Helicobacter pylori* components, 7-ketocholesterol and acetylsalicylic acid. Int. J. Mol. Sci..

[31] Matsuura E (2009). Autoimmunity, infectious immunity, and atherosclerosis. J. Clin. Immunol..

[32] Rodella, L. F. & Rezzani, R. Endothelial and vascular smooth cell dysfunction: a comprehensive appraisal. In *Artherogenesis* (ed. Parthasarathy, S.) under CC BY 3.0 license 105–134 (InTech, 2012).

[33] Kilic A (2006). Detection of cytomegalovirus and *Helicobacter pylori* DNA in arterial walls with grade III atherosclerosis by PCR. Pol. J. Microbiol..

[34] Kedzia A, Ciecierski M, Wierzbowska M, Kufel A, Kwapisz E (2010). Isolation of *Helicobacter pylori* from femoral or iliac atherosclerotic plaques. Acta Angiol..

[35] Raut SC, Patil VW, Dalvi SM, Bakhshi GD (2015). *Helicobacter pylori* gastritis, a presequeale to coronary plaque. Clin. Pract..

[36] Pasceri V (1998). Association of virulent *Helicobacter pylori* strains with ischemic heart disease. Circulation.

[37] Gunn M, Stephens JC, Thompson JR, Rathbone BJ, Samani NJ (2000). Significant association of CagA positive *Helicobacter pylori* strains with risk of premature myocardial infarction. Heart.

[38] Khodaii Z, Vakili H, Ghaderian SM, Najar RA, Panah AS (2011). Association of *Helicobacter pylori* infection with acute myocardial infarction. Coron. Artery Dis..

[39] Gonciarz W (2022). Antibodies towards TVLPVIFF amino acid sequence of TNFR receptor induced by *Helicobacter pylori* in patients with coronary heart disease. J. Clin. Med..

[40] Grebowska A (2006). Potential role of LPS in the outcome of *Helicobacter pylori* related diseases. Pol. J. Microbiol..

[41] Gonciarz W (2019). Autoantibodies to a specific peptide epitope of human Hsp60 (ATVLA) with homology to *Helicobacter pylori* HspB in *H. pylori*-related patients. APMIS.

[42] Vahdat K, Jafari SM, Pazoki R, Nabipour I (2007). Concurrent increased high sensitivity C-reactive protein and chronic infections are associated with coronary artery disease: A population-based study. Indian J. Med. Sci..

[43] Evrengul H (2007). Elevated homocysteine levels in patients with slow coronary flow: Relationship with *Helicobacter pylori* infection. Helicobacter.

[44] Akbas HS (2010). The assessment of carotid intima media thickness and serum paraoxonase-1 activity in *Helicobacter pylori* positive subjects. Lipids Health Dis..

[45] Polyzos SA, Kountouras J, Zavos C, Deretzi G (2011). The association between *Helicobacter pylori* infection and insulin resistance: A systematic review. Helicobacter.

[46] Rodriguez-Saona, L., Ayvaz, H., & Wehling, R. L. Infrared and Raman spectroscopy. In *Food Analysis. Food Science Text Series* (ed. Nielsen, S. S.) (Springer, 2017). 10.1007/978-3-319-45776-5_8.

[47] Zhou J, Wang Z, Sun S, Liu M, Zhang H (2001). Rapid method for detecting conformational changes during differentiation and apoptosis of HL60 cells by Fourier transform infrared spectroscopy. Biotechnol. Appl. Biochem..

[48] Deleris G, Petibois C (2003). Applications of FT-IR spectrometry to plasma contents analysis and monitoring. Vib. Spectrosc..

[49] Naumann, D. Infrared spectroscopy in microbiology. In *Encyclopedia of Analytical Chemistry*. (ed. Meyers, R.) 102–131 (Wiley, 2000).

[50] Dorling KM, Baker MJ (2013). Highlighting attenuated total reflection Fourier transform infrared spectroscopy for rapid serum analysis. Trends Biotechnol..

[51] Rakesh P, Charmi P, Rajesh KS (2014). Quantitative analytical applications of FTIR spectroscopy in pharmaceutical and allied areas. J. Adv. Pharm. Edu. Res..

[52] Mantsch, H. H. *Chapman. Infrared Spectroscopy of Biomolecules*. (Wiley, 1996) ISBN: 978-0-471-02184-1.

[53] Shen YC (2003). The use of Fourier-transform infrared spectroscopy for the quantitative determination of glucose concentration in whole blood. Phys. Med. Biol..

[54] Erukhimovitch V, Talyshinsky M, Souprun Y, Huleihel M (2006). FTIR spectroscopy examination of leukemia patients plasma. Vib. Spectrosc..

[55] Lechowicz L, Chrapek M, Gaweda J, Urbaniak M, Konieczna I (2016). Use of Fourier-transform infrared spectroscopy in the diagnosis of rheumatoid arthritis: A pilot study. Mol. Biol. Rep..

[56] Mordehai J (2004). Studies on acute human infections using FTIR microscopy and cluster analysis. Biopolymers.

[57] Steenbeke M (2020). Exploring the possibilities of infrared spectroscopy for urine sediment examination and detection of pathogenic bacteria in urinary tract infections. Clin. Chem. Lab. Med..

[58] Gonciarz W, Lechowicz Ł, Urbaniak M, Kaca W, Chmiela M (2021). Use of Fourier-Transform Infrared Spectroscopy (FT-IR) for monitoring experimental *Helicobacter pylori* infection and related inflammatory response in guinea pig model. Int. J. Mol. Sci..

[59] Gonciarz W, Lechowicz Ł, Urbaniak M, Kaca W, Chmiela M (2020). Attenuated Total Reflectance Infrared Spectroscopy (FTIR) and artificial neural networks applied to investigate quantitative changes of selected soluble biomarkers, correlated with *H. pylori* infection in children and presumable consequent delayed growth. J. Clin. Med..

[60] Peters AS (2017). Serum-infrared spectroscopy is suitable for diagnosis of atherosclerosis and its clinical manifestation. Vib. Spectrosc..

[61] Marzec KM (2015). Vascular diseases investigated ex vivo by using Raman, FT-IR and complementary methods. Pharmacol. Rep..

[62] Palombo F, Shen H, Benguigui LES, Kazarian SG, Upmacis RK (2009). Micro ATR-FTIR spectroscopic imaging of atherosclerosis: An investigation of the contribution of inducible nitric oxide synthase to lesion composition in ApoE-null mice. Analyst..

[63] Anderson OP (2006). Nanocrystalline diamond sensor targeted for selective CRP detection: An ATR-FTIR spectroscopy study. Anal. Bioanal. Chem..

[64] de Ruig, W.G. *Infrared Spectra of Monoacid Triglycerides with Some Applications to Fat Analysis*. Government Dairy Station, Leiden ISBN 90 220 0350 7. (Centre for Agricultural Publishing and Documentation, 1971).

[65] Cooper EA, Knutson K (1995). Fourier transform infrared spectroscopy investigations of protein structure. Pharm. Biotechnol..

[66] Fernández-Higuero JA, Salvador AM, Martín C, Milicua JCG, Arrondo JLR (2014). Human LDL structural diversity studied by IR spectroscopy. PLoS One..

[67] Daugherty A, Rateri DL (2002). T lymphocytes in atherosclerosis. The Yin-Yang of Th1 and Th2 influence on lesion formation. Circ. Res..

[68] Paraschuk DY (2005). Weak intermolecular charge transfer in the ground state of a π-conjugated polymer chain. J. Exp. Theor. Phys. Lett..

[69] Patel AF (2004). Serum triglycerides as a risk factor for cardiovascular diseases in the Asia-Pacific region. Asia Pacific Cohort Studies Collaboration. Meta-Analysis. Circulation.

[70] Ye X, Kong W, Zafar MI, Lu-Lu Ch (2019). Serum triglycerides as a risk factor for cardiovascular diseases in type 2 diabetes mellitus: A systematic review and meta-analysis of prospective studies. Cardiovasc. Diabetol..

[71] Lawlor DA (2008). The association of C-reactive protein and CRP genotype with coronary heart disease: findings from five studies with 4,610 cases amongst 18,637 participants. PLoS ONE.

[72] Bielański W, Konturek SJ (1996). New approach to ^13^C urea breath test capsule-based modification with low dose of ^13^C urea in the diagnosis of *Helicobacter pylori* infection. J. Physiol. Pharmacol..

[73] Rechcinski T, Chmiela M, Małecka-Panas E, Płaneta-Małecka I, Rudnicka W (1997). Serological indicators of *Helicobacter pylori* infection in adult dyspeptic patients and health blood donors. Microbiol. Immunol..

[74] Chmiela M (1998). Systemic humoral response to *Helicobacter pylori* in children and adults. Arch. Immunol. Ther. Exp..

[75] Tadeusiewicz R (2015). Neural networks as a tool for modeling of biological systems. Bio-Algorithms Med-Syst..

[76] Zurada J, Karwowski W, Marras WS (1997). A neural network-based system for classification of industrial jobs with respect to risk of low back disorders due to workplace design. Appl. Ergon..

[77] Ostrovski, G., Bellemare, M. G., Oord, A. & Munos, R. Count-based exploration with neural density models. In *International Conference on Machine Learning*. *PMLR.* 2721–2730. 10.48550/arXiv.1703.01310 (2017).

